# Low-Fat Nondairy Minidrink Containing Plant Stanol Ester Effectively Reduces LDL Cholesterol in Subjects with Mild to Moderate Hypercholesterolemia as Part of a Western Diet

**DOI:** 10.1155/2013/192325

**Published:** 2013-09-16

**Authors:** Maarit Hallikainen, Johan Olsson, Helena Gylling

**Affiliations:** ^1^Department of Clinical Nutrition, Institute of Public Health and Clinical Nutrition, University of Eastern Finland, P.O. Box 1627, 70211 Kuopio, Finland; ^2^Foodfiles AB, Dag Hammarskjöldsväg 36B, 751 83 Uppsala, Sweden; ^3^Department of Medicine, Division of Internal Medicine, University of Helsinki, P.O. Box 700, 00029 Helsinki, Finland

## Abstract

The cholesterol-lowering efficacy of plant stanol ester (STAEST) added to fat- or milk-based products is well documented. However, their efficacy when added to nondairy liquid drinks is less certain. Therefore, we have investigated the cholesterol-lowering efficacy of STAEST added to a soymilk-based minidrink in the hypercholesterolemic subjects. In a randomized, double-blind, placebo-controlled parallel study, the intervention group (*n* = 27) consumed 2.7 g/d of plant stanols as the ester in soymilk-based minidrink (65 mL/d) with the control group (*n* = 29) receiving the same drink without added plant stanols once a day with a meal for 4 weeks. Serum total, LDL, and non-HDL cholesterol concentrations were reduced by 8.0, 11.1, and 10.2% compared with controls (*P* < 0.05 for all). Serum plant sterol concentrations and their ratios to cholesterol declined by 12–25% from baseline in the STAEST group while the ratio of campesterol to cholesterol was increased by 10% in the controls (*P* < 0.05 for all). Serum precursors of cholesterol remained unchanged in both groups. In conclusion, STAEST-containing soymilk-based low-fat minidrink consumed once a day with a meal lowered LDL and non-HDL cholesterol concentrations without evoking any side effects in subjects consuming normal Western diet. The clinical trial registration number is NCT01716390.

## 1. Introduction

It is known that the best way to reduce the risk of coronary artery disease (CAD) is to lower the low-density lipoprotein (LDL) cholesterol level. It has been estimated that each 1% reduction in LDL cholesterol achieves a 1% reduction in the risk of CAD [1]. Phytosterols have attracted considerable interest as cholesterol-lowering agents since the 1950s [2] due to their ability to reduce the serum LDL cholesterol level by interfering with cholesterol absorption. In a recent meta-analysis, LDL cholesterol values were reduced by 9% with a 2 g daily dose of plant stanols, and furthermore increasing the daily intake of plant stanols was found to dose-dependently reduce LDL cholesterol [3]. Most of the studies included in the plant stanol ester (STAEST) meta-analysis have been performed with solid food format, and only in four studies out of 61 has the plant stanol been in liquid dairy and one in a nondairy form [3]. 

The EFSA NDA Panel concluded in its heath claim dossier that STAEST at a daily intake of 3 g plant stanols (range of 2.7 g to 3.3 g) in matrices approved by Regulation (EC) No 376/2010 (yellow fat spreads, dairy products, mayonnaise, and salad dressings) can lower LDL cholesterol by 11.4% (95% CI: 9.8–13.0) and STAEST added to foods such as margarine-type spreads, mayonnaise, salad dressings, and dairy products such as milk, yoghurts including low-fat yoghurts, and cheese has been shown consistently to lower blood LDL cholesterol levels, but the extent of the cholesterol-lowering effect of plant sterols/stanols added to other food formats is less well established [4]. From a theoretical point of view one could speculate that there might be no difference in the cholesterol-lowering effect of plant stanols or plant sterols based dairy versus nondairy products, but nonetheless the impact of the different food formats and background diets needs to be confirmed experimentally. 

To this end, the aim of this investigation was to evaluate the efficacy of plant stanols (2.7 g/d) consumed once a day as a low-fat STAEST soymilk-based minidrink to lower LDL cholesterol level as well as its safety in a placebo-controlled, randomized, parallel study in individuals with mild to moderate hypercholesterolemia. 

## 2. Subjects and Methods

### 2.1. Subjects

The subjects were recruited by advertisements placed in local papers in Uppsala, Sweden, from the pool of volunteers at Foodfiles (former Good Food Practice), Uppsala, Sweden, and via webpages. Inclusion criteria were as follows: age 25–65 years, serum total cholesterol level 5.2–8.5 mmol/L, and normal liver, kidney, and thyroid function. Exclusion criteria were as follows: severe obesity (BMI > 35.0 kg/m^2^), consumption of lipid lowering medication or other drugs affecting lipid metabolism, consumption of plant stanol or plant sterol products, history of unstable CAD, myocardial infarction or unstable angina pectoris within the previous 6 months, transient ischemic attack or stroke within 1 year, diabetes, malignant disease, being pregnant or lactating, alcohol or substance abuse, or lack of suitability for participation in the trial. On the basis of these criteria, 90 subjects were screened, and of these 61 subjects were ultimately included in the intervention. Two subjects withdrew from the control group due to an adverse event and three subjects from the STAEST group due to an adverse event, unwillingness to continue or a major protocol violation (8 kg weight reduction). Thus, a total of 56 subjects completed the study. 

Five subjects had hypertension, for which two subjects were taking diuretics and four were receiving angiotensin converting enzyme or angiotensin receptor blocking agents. One subject was using hormone replacement therapy. The subjects were requested to keep their medication, weight, lifestyle, and dietary habits unchanged during the study. 

All subjects gave their written informed consent for the intervention. The intervention was performed according to the principles of the Declaration of Helsinki. The study protocol was approved by the Ethical Regional Review Board in Uppsala, Sweden.

### 2.2. Study Design

The intervention was a randomized, double-blind, placebo-controlled trial with parallel design. It contained a 2-week screening phase and a 4-week intervention. During the 2-week screening phase, the subjects were screened for inclusion and exclusion criteria. After screening, the subjects were randomized according to a randomization treatment list generated by an independent statistician. The STAEST group (*n* = 27) consumed a soy-based minidrink with added plant stanol esters and the control group (*n* = 29) the same minidrink without added plant stanols daily for 4 weeks with either lunch or dinner. The baseline characteristics of the subjects are shown in Table 1. 

Samples for routine laboratory measurements were taken to ensure normal health status before the entry and at the end of the intervention. In addition, diseases, possible medications, and use of vitamins or other nutrient supplements were recorded at all visits and possible adverse events at visits of weeks 3 and 4. Samples for laboratory measurements were taken at the beginning (weeks −1 and 0) and at the end of the intervention (weeks 3 and 4) for lipids and on weeks 0 and 4 to analyze serum concentrations of serum squalene and noncholesterol sterols. For the lipids, the mean values of weeks −1 and 0 and weeks 3 and 4 were used for in the statistical analyses. 

### 2.3. Diet

The soy-based minidrinks were provided by Raisio Nutrition Ltd. (Raisio, Finland). The test minidrinks (65 mL) contained energy 151 kJ (36 kcal), protein 1.1 g, carbohydrates 2.1 g, absorbable fats 2.5 g, and fiber 0.5 g. The subjects were advised to continue with their habitual diet and take the test minidrink (65 mL/d) directly after lunch or dinner. The analyzed daily amount of plant stanols was 2.8 g (27.9% campestanol and 72.1% sitostanol) in the STAEST minidrink which amounted to 2.7 g taking into account the compliance reported by the subjects. To verify the compliance, the subjects were asked to fill in a form detailing the use of the test minidrinks including timing (date, precise clock time, lunch, or dinner) in a study diary on every day for the 4 weeks of the trial. They were also asked to fill in any failures to take the product and also if they did not consume the total contents of the test mini-drink. 

The diet was monitored from a 3-d (2 weekdays and 1 weekend day) food diary kept at baseline and at the end of the intervention. The nutrients in the food diaries were calculated by using Dietist XP (version 3, Bromma, Sweden). 

### 2.4. Clinical and Laboratory Measurements

Body weight was measured with a digital scale and height using a stadiometer. 

All blood samples were collected after 12 h overnight fast. Laboratory samples were analyzed with routine standardized methods at the Aleris Medilab, Täby, Sweden. 

Serum total and HDL cholesterol and serum triglycerides were analyzed with an Architect c16000 Clinical Chemistry Analyzer (Abbott Diagnostics Division, USA) using enzymatic methods (Abbott Scandinavia AB, Solna, Sverige). The LDL cholesterol level was calculated by the Friedewald equation.

Serum cholesterol, cholesterol precursors (squalene, cholestenol, desmosterol, and lathosterol), plant sterols (sitosterol, campesterol, and avenasterol), plant stanol (sitostanol), and cholestanol were quantified from nonsaponifiable serum material by capillary gas-liquid chromatography (GLC) (Agilent 6890N Network GC System, Agilent Technologies, Wilmington, DE, USA) equipped with a 50 m long Ultra 2 capillary column (5% phenyl-methyl siloxane) (Agilent Technologies, Wilmington, DE, USA) [5]. Serum measurements were expressed as concentrations (*μ*g/dL), but also in terms of 10^2^ × mmol/mol of cholesterol (called the ratio in the text) by dividing the concentrations with the cholesterol value of the same GLC run in order to eliminate the impact of changes in the concentrations of sterol transporting lipoproteins, mainly LDL. The concentrations of serum cholesterol precursors and especially their ratios to cholesterol reflect whole-body cholesterol synthesis, and the concentrations of plant sterols and cholestanol and especially their ratios to cholesterol reflect cholesterol absorption [6, 7]. 

### 2.5. Statistical Analyses

All statistical analyses were performed with the SPSS for Windows 19.0 statistics program (SPSS, Chicago, IL, USA). 

Normality and homogeneity of variance assumptions were evaluated before conducting detailed statistical analyses. Univariate analysis of variance was used to compare the baseline values and the changes between groups. The analysis of variance for repeated measurements (GLM) was used to analyze the interaction of time and groups, effect of gender, and changes over time in between-group comparisons followed by post hoc comparisons with Bonferroni corrections. The nutrient intakes (protein and fiber) that significantly differed between the study groups or significantly changed within the study group were included as covariates in the ANCOVA analyses. Variables not normally distributed even after logarithmic transformation or nonhomogenous in variance or non-continuous were tested with the Mann-Whitney *U*-test or Fisher exact test. A *P* value of <0.05 was considered statistically significant. The results are given as means ± SEM.

## 3. Results

### 3.1. Baseline

The baseline characteristics are shown in Table 1. The mean age of the whole study population was 54.8 ± 1.2 yrs with a range from 30 to 66 yrs., BMI 24.1 ± 0.4 kg/m^2^, and serum cholesterol 6.5 ± 0.1 mmol/L. There were no significant differences in any of the clinical variables or in the frequencies of diseases or medications (data not shown) between the study groups, except that serum creatinine was lower in the STAEST group in comparison with the controls. 

### 3.2. Intervention

#### 3.2.1. Weight and BMI

Weight and BMI did not change significantly during the intervention (Table 1).

#### 3.2.2. Feasibility of the Diet

The compliance with the consumption of the test minidrinks was good. The minidrinks were consumed on average by 97% of the intended amount, resulting in an estimated mean daily intake of plant stanols of 2.7 g. The intake of protein and fiber from the diet decreased in the STAEST group with the latter differing significantly from the controls (Table 2). The intake of fat increased similarly from baseline in both study groups (*P* < 0.05), but there were no significant changes in intakes of other nutrients in either of the study group during the intervention. Two subjects in each group reported gastrointestinal discomfort.

#### 3.2.3. Safety Clinical Chemistry

The values of hemoglobin and hematocrit, number of erythrocytes and the counts of blood leucocytes similarly and significantly decreased, and mean corpuscular hemoglobin and mean corpuscular hemoglobin concentrations increased in both study groups during the intervention, but all values remained within the reference values (Table 1). Amino alanine aminotransferase, gamma-glutamyltransferase, and thyroid stimulating hormone levels did not change during the intervention. Serum creatinine similarly decreased from baseline in both groups when the baseline value was taken into account in the statistical analysis. However, the decrease was not statistically significant after adjustment for the changes in the nutrient intakes. 

The plasma glucose level similarly decreased from baseline in both groups (*P* < 0.01). The serum high-sensitive C-reactive protein concentration remained unchanged during the intervention.

#### 3.2.4. Serum Lipids

**Figure 1:**
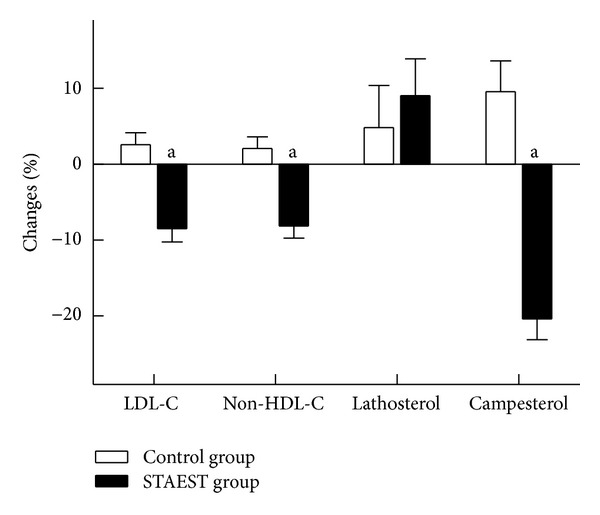
The percentage changes in LDL cholesterol (LDL-C) and non-HDL cholesterol (non-HDL-C) concentrations and the ratios of serum lathosterol and campesterol to cholesterol from baseline in the control (*n* = 29) and STAEST groups (*n* = 27 except *n* = 26 for LDL-C and non-HDL-C). ^a^ *P* < 0.05 significantly different from control group.

In the STAEST group, the analysis of serum lipids could be performed from 26 subjects. Serum total, LDL, and non-HDL cholesterol concentrations and LDL/HDL ratio were reduced by 6.4, 8.5, 8.2, and 6.4% compared with the baseline values in the STAEST group (*P* < 0.05 for all, Table 1, Figure 1). The respective reductions were 8.0, 11.1, 10.2, and 9.0% compared with controls (*P* < 0.05 for all). After adjustment for changes in protein and fiber intakes, the intervention value of LDL and non-HDL cholesterol remained statistically significantly different between the groups (*P* < 0.05), but total cholesterol only tended to differ between the groups (*P* = 0.072). The differences in the percentage changes in serum total, LDL, and non-HDL cholesterol concentrations and LDL/HDL ratio remained significant after the adjustment for the changes in protein and fiber intakes. The HDL cholesterol and serum triglyceride concentrations remained unchanged during the intervention. 

#### 3.2.5. Cholesterol Precursors, Plant Sterols, and Cholestanol

There were no significant changes in serum squalene, cholestenol, or lathosterol (Figure 1) concentrations or their ratios to cholesterol during the study (Table 3). The ratio of serum desmosterol to cholesterol, but not its concentration, increased similarly in both groups (*P* < 0.05). 

Serum campesterol (Figure 1), sitosterol, and avenasterol concentrations and their ratios to cholesterol were significantly reduced by 12–25% and 7–20%, respectively, in the STAEST group during the study (Table 3). The serum campesterol to cholesterol ratio increased by 10% in the controls (Figure 1), but no other significant changes were observed in plant sterol concentrations or in their ratios to cholesterol (Table 3). The differences in the changes in serum campesterol, sitosterol, and avenasterol concentrations and their ratios to cholesterol were from −28 to −38% and from −21 to −47%, respectively, in the STAEST group in comparisons with controls (*P* < 0.05). Adjustments taking into account the changes in protein and fiber intakes did not alter these results. Serum cholestanol concentrations and ratios to cholesterol were similarly decreased by 0.4–9% and 1–4% in both study groups (*P* < 0.05). After adjustment with the changes in the nutrient intakes, the serum cholestanol concentration declined significantly only in the STAEST group.

In the STAEST group, the serum sitostanol concentration and the ratio to cholesterol were increased by 38% and 47% compared with the baseline values and compared with controls, but its concentration during the intervention remained low, below 45 *μ*g/dL (Table 3). 

## 4. Discussion

The novel finding was that consumption of 2.7 g/d of plant stanols as stanol ester in soy-based minidrink once a day lowered the LDL cholesterol level by 0.51 mmol/L corresponding to 11.1% reduction compared with controls in subjects consuming a habitual Western diet. In addition, serum total and non-HDL cholesterol concentrations and the LDL/HDL ratio were significantly reduced, and the product was welltolerated. There is only one previous study investigating the addition of STAEST (2 g/d of plant stanols) to a low-fat nondairy minidrink, that is, soymilk-based minidrink in a Thai population where it achieved a 9% LDL cholesterol reduction [8]. With regard to low-fat dairy products, there is one earlier study examining a low-fat dairy minidrink containing STAEST (2 g/d of plant stanols) [9] and four studies with a similar low-fat dairy minidrink but with plant sterol ester (1.6–3 g/d of plant sterols) [10–13]. The LDL cholesterol lowering efficacy in these studies was 8–10%. It was also apparent that the frequency of the ingestion of low-fat dairy or nondairy minidrinks did not affect the results; the LDL cholesterol lowering efficacy was the same whether the minidrink was consumed once a day [8–10, 12, 13] or twice a day [11] either with the meal or just after the meal. The LDL cholesterol reduction was also of the same magnitude as encountered in the previous STAEST and plant sterol ester studies with fat-based spreads, salad dressings or mayonnaises, or dairy products consumed two to three times a day with the meal [3]. 

The randomization of the subjects was successful, and there were no significant differences in the background variables at baseline, except that serum creatinine was lower in the STAEST group as compared with the controls. The changes in blood count and serum creatinine and plasma glucose concentrations were similar in both groups during the intervention, and the values remained within the reference values. The intake of protein and fiber decreased in the STAEST group, and the intake of fiber differed significantly from the controls. When the changes in the intake of protein and fiber were taken into account in the analyses as covariates, they did not alter the results except that the intergroup difference in the intervention value of serum total cholesterol did not quite achieve statistical significance. 

The timing of the consumption of plant stanol and sterol-containing products has been postulated to play a crucial role in the cholesterol-lowering efficacy. In a previous study, when the participants consumed the plant sterol drink with a meal, a significantly greater reduction in LDL cholesterol was reported as compared to drinking it before breakfast or on an empty stomach [10]. In the latter case, the minidrink appeared to be rapidly emptied from the stomach without triggering any contraction of the gallbladder, and thus bile acids and biliary cholesterol were not sufficiently released into the small intestine [14]. The lack of bile acids could interfere with the proper formation of micelles, which are formed through the plant stanol-cholesterol interaction. It has been documented that plant stanols interfere with the micellar solubility of cholesterol [15]; that is, less cholesterol is taken up by the intestinal NPC1L1 receptor. Similar results have been demonstrated with plant sterols such that ultimately the amount of meal-derived cholesterol becomes reduced first in chylomicrons and subsequently in plasma [16]. 

It is known that serum cholesterol precursors reflect the whole-body cholesterol synthesis, and serum plant sterols and cholestanol reflect the cholesterol absorption efficiency in different populations under steady state conditions [6, 7, 17] and during STAEST consumption [18, 19]. In general, cholesterol absorption and synthesis are counterbalanced such that, when cholesterol absorption is reduced, then the cholesterol synthesis is upregulated. However, the magnitude of the upregulation of cholesterol synthesis evoked by the plant sterol/stanol-induced cholesterol malabsorption seems to vary. In most of the STAEST studies, in which spread (e.g., [18–21]) or low-fat yoghurt [22] was consumed two to three times a day with a meal, cholesterol synthesis was increased [18–22]. However, in the present investigation when STAEST in soy-based minidrink was consumed once a day with a meal, no significant changes were found in serum cholesterol precursor concentrations or in their ratios to cholesterol. Similar results were obtained in subjects with type 1 diabetes without or with statin treatment during trials with STAEST spread [23, 24] and plant sterol ester added to mini-yoghurt drink [11–13] or soymilk [25] consumed once [12, 13] or twice [11] or three times [25] a day with a meal. The results with plant sterol ester in drinkable products are not, however, totally consistent. In one study, the addition of plant sterol ester to semiskimmed vegetable fat-enriched milk significantly increased the serum lathosterol to cholesterol ratio when the milk was consumed twice a day with a meal [26]. With respect to the frequency of plant sterol consumption, the cholesterol fractional synthesis rate tended to be greater when plant sterol-containing spread was consumed three times versus once a day [27]. Timing of administration has also been examined; in one study cholesterol fractional synthesis significantly increased when plant sterol-containing low-fat yoghurt was consumed with a meal [28]. Accordingly, whether or not cholesterol synthesis is upregulated does not seem to depend on the food format (solid/liquid/fat content) or frequency of timing, and the ultimate reason remains open. 

In line with the STAEST studies with spread (e.g., [21, 29]) or low-fat yoghurt [22], in the present intervention, STAEST minidrink significantly decreased serum plant sterol concentrations and their ratios to cholesterol; for example, the control-related serum campesterol to cholesterol ratio was decreased by 30%. The reduction in serum plant sterols during STAEST consumption reflects compliance. In previous studies from our group where the plant stanol dose was 2.4–3 g/d resembling the present dose, the relative serum campesterol reduction has varied from 35% to 43% [18, 19, 21, 29]. In the present study, in the controls the serum campesterol to cholesterol ratio increased by 10% from baseline. This is probably due to greater intake of vegetable oil during the intervention period compared to the baseline even though no significant increase in the intakes of the monounsaturated or polyunsaturated fatty acids was detected. The serum sitostanol concentration was increased by 11.5 *μ*g/dL corresponding to a relative increase of 38%. In our earlier studies, the mean relative increase in serum sitostanol concentration has varied from 67 to 96% with a similar plant stanol dose. Accordingly, in the present study, the reduction in serum plant sterols and the increase in serum sitostanol were evidence of acceptable compliance. 

Cholestanol is a 5*α*-saturated derivative of cholesterol produced by the liver. It has been shown earlier in trials where subjects were given plant stanols that serum cholestanol concentration and its ratio to cholesterol correlated with the fractional cholesterol absorption [18, 19]. In the present study, after the adjustment for the changes in protein and fiber intakes, the serum cholestanol concentration was significantly reduced only in the STAEST group. In some earlier studies, the results of cholestanol concentration and its ratio to cholesterol have varied for unknown reasons (e.g., [23, 29]).

In conclusion, the intake of 2.7 g/day of plant stanols as an ester once a day in a low-fat soy-based minidrink effectively reduced serum LDL cholesterol concentrations by 11.1% in comparison to controls consuming a regular Western diet. Furthermore, the products did not cause any major side effects. 

## Figures and Tables

**Table 1:** Clinical variables during the study.

	Control group (*n* = 29)		STAEST group (*n* = 27)		*P* ^a^
	Baseline	Intervention	Baseline	Intervention	
*N* (M/W)	7/22		4/23		0.506^b^
Age (y)	54.2 ± 1.8		55.3 ± 1.6		0.837^b^
Weight (kg)	70.1 ± 1.8	70.0 ± 1.8	66.3 ± 1.8	66.0 ± 1.8	0.696
BMI (kg/m^2^)	24.5 ± 0.5	24.5 ± 0.5	23.7 ± 0.5	23.6 ± 0.5	0.696
Blood leukocytes (10^9^/L)^c^	5.10 ± 0.26	4.64 ± 0.25	5.26 ± 0.20	4.99 ± 0.21	0.681
Blood erythrocytes (10^12^/L)^c^	4.70 ± 0.07	4.59 ± 0.07	4.54 ± 0.07	4.48 ± 0.06	0.841
Hemoglobin (g/L)^c^	140.4 ± 2.1	138.2 ± 1.9	137.7 ± 1.9	136.3 ± 1.9	0.635
Hematocrit (%)^c^	0.42 ± 0.01	0.41 ± 0.0	0.41 ± 0.01	0.40 ± 0.0	0.453
MCV (fl)	88.9 ± 0.7	89.1 ± 0.8	90.3 ± 0.8	89.5 ± 0.8	0.188
MCH (pg)^c^	29.9 ± 0.3	30.2 ± 0.3	30.3 ± 0.3	30.5 ± 0.3	0.914
MCHC (g/L)^c^	336.9 ± 1.6	338.9 ± 1.7	336.1 ± 1.5	340.5 ± 1.8	0.301
Blood thrombocytes (10^9^/L)	255.4 ± 6.9	257.7 ± 7.7	268.0 ± 10.6	258.0 ± 9.0	0.075
ALT(*μ*kat/L)	0.46 ± 0.05	0.47 ± 0.06	0.45 ± 0.03	0.46 ± 0.04	0.800
GGT (U/L)	0.42 ± 0.05	0.41 ± 0.04	0.38 ± 0.05	0.41 ± 0.06	0.895
Creatinine (*μ*mol/L)^c^	70.8 ± 1.7	68.9 ± 1.9	65.6 ± 1.8^d^	64.2 ± 1.8	0.788
Thyroid stimulating hormone (mlE/L)	1.77 ± 0.13	1.72 ± 0.13	1.81 ± 0.17	1.93 ± 0.19	0.098
hs-CRP (mg/L)	1.54 ± 0.34	1.56 ± 0.34	1.42 ± 0.35	1.54 ± 0.40	0.838
Plasma glucose (mmol/L)^c^	5.04 ± 0.09	4.83 ± 0.08	5.07 ± 0.10	4.95 ± 0.12	0.573
Serum total cholesterol (mmol/L)^f^	6.54 ± 0.16	6.64 ± 0.18	6.49 ± 0.20	6.03 ± 0.14^d,e^	<0.001
LDL cholesterol (mmol/L)^f^	4.44 ± 0.16	4.54 ± 0.17	4.35 ± 0.18	3.94 ± 0.13^d,e^	<0.001
HDL cholesterol (mmol/L)^f^	1.59 ± 0.08	1.59 ± 0.08	1.71 ± 0.08	1.67 ± 0.07	0.421
Serum triglycerides (mmol/L)^f^	1.14 ± 0.11	1.12 ± 0.10	0.95 ± 0.06	0.93 ± 0.07	0.558
Non-HDL cholesterol (mmol/L)^f^	4.95 ± 0.18	5.05 ± 0.19	4.78 ± 0.19	4.36 ± 0.15^d,e^	<0.001
Ratio LDL/HDL cholesterol^f^	3.02 ± 0.21	3.09 ± 0.21	2.68 ± 0.17	2.50 ± 0.16^d,e^	0.003

Values shown are means ± SEM. ALT: alanine aminotransferase; GGT: gamma-glutamyltransferase; hs-CRP: high sensitive C-reactive protein; MCH: mean corpuscular hemoglobin; MCHC: mean corpuscular hemoglobin concentration; MCV: mean corpuscular volume; and STAEST: plant stanol ester.

^a^Group by time interaction (repeated measured variance of analysis (general linear model)).

^b^Difference between the groups: gender (Fisher exact test) and age (univariate analysis of variance (general linear model)).

^c^ *P* < 0.05, significant change over time.

^d^ *P* < 0.05 significantly different from control.

^e^ *P* < 0.05 significantly different from baseline.

^f^Control (*n* = 29), STAEST (*n* = 26).

**Table 2:** Nutrient intakes during the intervention.

	Control group (*n* = 29)		STAEST group (*n* = 27)		*P* ^a^
	Baseline	Intervention	Baseline	Intervention	
Energy (MJ/d)	7.4 ± 0.3	7.9 ± 0.4	7.3 ± 0.3	7.0 ± 0.3	0.267
Fat (% of energy)^b^	32.5 ± 1.1	34.6 ± 1.3	34.4 ± 1.2	37.2 ± 1.6	0.668
SFA (% of energy)	12.4 ± 0.5	13.1 ± 0.6	13.0 ± 0.7	13.9 ± 0.8	0.862
MUFA (% of energy)	12.4 ± 0.5	12.9 ± 0.5	12.7 ± 0.5	14.0 ± 0.8	0.340
PUFA (% of energy)	4.8 ± 0.3	5.5 ± 0.3	5.0 ± 0.3	5.4 ± 0.3	0.741
Proteins (% of energy)	15.9 ± 0.5	16.1 ± 0.5	16.3 ± 0.5	14.9 ± 0.4^c^	0.029
Carbohydrates (% of energy)	42.2 ± 1.2	40.8 ± 1.0	40.6 ± 1.1	39.2 ± 1.3	0.766
Alcohol (% of energy)	3.6 ± 0.9	2.8 ± 0.6	3.2 ± 0.6	4.0 ± 0.8	0.215
Total fiber (g/d)	20.0 ± 1.1	20.6 ± 1.0	20.6 ± 1.4	17.7 ± 1.1^c,d^	0.032
Total fiber (g/MJ)	2.8 ± 0.2	2.7 ± 0.2	2.8 ± 0.2	2.5 ± 0.1	0.362

All values are means ± SEM. Intervention values do not contain the nutrient intake from the test minidrink. MUFA: monounsaturated fatty acids; PUFA: polyunsaturated fatty acids; SFA: saturated fatty acids; and STAEST: plant stanol ester.

Baseline values did not differ significantly between the groups for any of the variables.

^a^Group by time interaction (repeated measured variance of analysis (general linear model)).

^b^ *P* < 0.05, significant change over time.

^c^ *P* < 0.05 significantly different from baseline.

^d^ *P* < 0.05 significantly different from control.

**Table 3:** Serum squalene and noncholesterol sterols during the intervention.

	Control group (*n* = 29)		STAEST group (*n* = 27)		*P* ^a^
	Baseline	Intervention	Baseline	Intervention	
Squalene (*μ*g/dL)	41.0 ± 2.4	42.8 ± 3.8	43.7 ± 2.8	40.4 ± 3.2	0.412
Cholestenol (*μ*g/dL)	45.8 ± 3.6	45.9 ± 3.0	43.9 ± 3.2	43.7 ± 2.6	0.686
Desmosterol (*μ*g/dL)	178.0 ± 6.7	183.8 ± 8.6	177.7 ± 6.4	176.2 ± 6.8	0.411
Lathosterol (*μ*g/dL)	258.6 ± 22.8	261.7 ± 21.3	262.9 ± 22.9	260.0 ± 21.5	0.660
Cholestanol (*μ*g/dL)^b^	371.8 ± 19.5	370.3 ± 22.5	357.6 ± 25.5	312.9 ± 14.9	0.081
Campesterol (*μ*g/dL)	650.8 ± 51.1	713.4 ± 59.1	588.7 ± 91.2	417.5 ± 54.9^c,d^	<0.001
Sitosterol (*μ*g/dL)	371.4 ± 30.6	393.2 ± 35.8	338.4 ± 39.8	249.0 ± 23.1^c,d^	<0.001
Sitostanol (*μ*g/dL)	31.9 ± 0.8	31.8 ± 0.9	30.5 ± 1.1	41.9 ± 1.7^c,d^	<0.001
Avenasterol (*μ*g/dL)	101.1 ± 6.4	104.6 ± 7.5	94.7 ± 9.3	76.5 ± 5.0^c,d^	0.011
Ratios to cholesterol (10^2^ mmol/mol of cholesterol)					
Squalene	17.6 ± 1.1	17.8 ± 1.2	19.4 ± 1.3	19.0 ± 1.3	0.821
Cholestenol	19.9 ± 1.7	19.9 ± 1.5	19.7 ± 1.4	20.8 ± 1.2	0.379
Desmosterol^b^	76.0 ± 2.4	77.6 ± 2.5	79.3 ± 3.2	83.4 ± 2.9	0.198
Lathosterol	111.9 ± 9.8	112.2 ± 8.7	118.0 ± 10.3	123.1 ± 9.6	0.506
Cholestanol^b^	158.4 ± 7.4	155.7 ± 7.4	153.7 ± 6.2	146.7 ± 5.1	0.379
Campesterol	276.0 ± 18.7	299.1 ± 20.7^c^	244.3 ± 27.7	193.3 ± 22.5^c,d^	<0.001
Sitosterol	157.6 ± 11.4	164.3 ± 12.5	142.5 ± 11.6	116.3 ± 9.6^c,d^	<0.001
Sitostanol	13.7 ± 0.3	13.6 ± 0.3	13.4 ± 0.2	19.7 ± 0.6^c,d^	<0.001
Avenasterol	42.9 ± 2.3	43.7 ± 2.4	40.2 ± 2.7	35.9 ± 2^c,d^	0.029

Values shown are means ± SEM. STAEST: plant stanol ester.

^a^Group by time interaction (repeated measured variance of analysis (general linear model)).

^b^ *P* < 0.05, significant change over time.

^c^ *P* < 0.05 significantly different from baseline.

^d^ *P* < 0.05 significantly different from control.
